# Clinical‐effectiveness of DREAMS START an intervention for with people with dementia and sleep disturbance and their caregivers ‐ A single masked, phase 3, parallel arms superiority randomised controlled trial

**DOI:** 10.1002/alz.094661

**Published:** 2025-01-09

**Authors:** Penny Rapaport, Sarah Amador, Julie Barber, Mariam M Adeleke, Lucy A Webster, Sube Banerjee, Georgina Charlesworth, Christopher Clarke, Colin A Espie, Simon D Kyle, Malgorzata Raczek, Zuzana Walker, Gill Livingston

**Affiliations:** ^1^ University College London, London United Kingdom; ^2^ UCL, london United Kingdom; ^3^ University of Nottingham, Nottingham United Kingdom; ^4^ North East London Foundation Trust, London United Kingdom; ^5^ Tees Esk and Wear Valley NHS Foundation Trust, Darlington United Kingdom; ^6^ University of Oxford, Oxford United Kingdom; ^7^ Brighton and Sussex Medical School (BSMS), brighton United Kingdom; ^8^ Sussex Partnership NHS Foundation Trust, Horsham United Kingdom; ^9^ Division of Psychiatry, University College London, London United Kingdom; ^10^ Essex Partnership University NHS Foundation Trust, Wickford United Kingdom; ^11^ Camden and Islington NHS Foundation Trust, London United Kingdom

## Abstract

**Background:**

Sleep disturbance is common and distressing for people with dementia, with no known safe, effective treatments. We previously developed and delivered DREAMS‐START (Dementia RElAted Manual for Sleep; STrAtegies for RelaTives), a multimodal non‐pharmacological intervention, and demonstrated feasibility and acceptability. This randomised controlled trial (RCT) aimed to establish whether DREAMS‐START is clinically‐effective in reducing sleep disturbances in people with dementia at home after 8 months compared to usual care.

**Methods:**

We conducted a two‐arm, multi‐centre, parallel arms, superiority RCT with masked outcome assessment, recruiting dyads of people with dementia and sleep disturbance, and their family caregivers, in English community settings. Those meeting inclusion criteria were randomised (1:1) to DREAMS‐START or usual treatment. DREAMS‐START is a six‐session, manualised intervention delivered by supervised, non‐clinical graduates. Strategies were tailored and included routine, comfort, increasing light exposure, relaxation, and activity. Outcomes were collected at baseline, 4 months, and 8 months. The primary outcome was the Sleep Disorders Inventory (SDI) score at 8 months and analyses were intention to treat. Secondary outcomes for people with dementia included quality of life, daytime sleepiness, and neuropsychiatric symptoms and for family caregivers ‐ quality of life, sleep disturbance, mood, and burden.

**Results:**

Between February 2021 and March 2023, 377 participant dyads were randomised, 189 were allocated to usual treatment and 188 to intervention. Mean age of participants with dementia was 79.4 years (SD 9.0). 206 (54.6%) were women. Mean SDI score at 8 months was lower in the intervention arm versus usual treatment (15.16 [SD 12.77], n = 159, vs 20.34 [16.67], n = 163]; adjusted difference in means ‐4.70 (95% CI ‐7.65 to ‐1.74; p = 0.002). At 8 months carer sleep (difference in means 0.57 [95% CI 0.10 to 1.05]) and carer anxiety (difference in means ‐0.86 [95% CI ‐1.71 to ‐0.01]), were significantly lower in the intervention group than usual treatment.

**Conclusion:**

To our knowledge, this is the first and largest fully powered RCT of a multicomponent non‐pharmacological intervention that improves sleep in people living at home with dementia and their caregivers, with sustained effectiveness beyond delivery. DREAMS‐START has potential to be delivered at scale in health services.

